# Clonal Diversity and Epidemiological Characteristics of ST239-MRSA Strains

**DOI:** 10.3389/fcimb.2022.782045

**Published:** 2022-03-25

**Authors:** Marwa I. Abd El-Hamid, Alaa H. Sewid, Mohamed Samir, Wael A. H. Hegazy, Mosa M. Bahnass, Rasha A. Mosbah, Doaa M. Ghaith, Eman Khalifa, Hazem Ramadan, Walaa A. Alshareef, Hanan M. Alshareef, Mohammed M. Ghoneim, Mohammad M. Al-Sanea, Mahmoud M. Bendary

**Affiliations:** ^1^ Department of Microbiology, Faculty of Veterinary Medicine, Zagazig University, Zagazig, Egypt; ^2^ Department of Zoonoses, Faculty of Veterinary Medicine, Zagazig University, Zagazig, Egypt; ^3^ Department of Microbiology and Immunology, Faculty of Pharmacy, Zagazig University, Zagazig, Egypt; ^4^ Department of Animal Medicine (Infectious Disease), Faculty of Veterinary Medicine, Zagazig University, Zagazig, Egypt; ^5^ Fellow Pharmacist, Zagazig University Hospital, Zagazig, Egypt; ^6^ Department of Clinical and Chemical Pathology, Faculty of Medicine, Cairo University, Cairo, Egypt; ^7^ Department of Microbiology, Faculty of Veterinary Medicine, Matrouh University, Matrouh, Egypt; ^8^ Hygiene and Zoonoses Department, Faculty of Veterinary Medicine, Mansoura University, Mansoura, Egypt; ^9^ Department of Microbiology, Faculty of Pharmacy, October 6 University, 6th of October, Egypt; ^10^ Department of Pharmacy Practice, Faculty of Pharmacy, University of Tabuk, Tabuk, Saudi Arabia; ^11^ Department of Pharmacy Practice, College of Pharmacy, Al Maarefa University, Ad Diriyah, Saudi Arabia; ^12^ Pharmaceutical Chemistry Department, College of Pharmacy, Jouf University, Sakaka, Saudi Arabia; ^13^ Department of Microbiology and Immunology, Faculty of Pharmacy, Port Said University, Port Said, Egypt

**Keywords:** MRSA, ST239, MDR, genetic background, evolution

## Abstract

Methicillin-resistant *Staphylococcus aureus* (MRSA) is a major pathogen associated with severe morbidity and mortality and poses a significant threat to public health worldwide. The genetic diversity based on sequence types of MRSA strains was illustrated in previous studies; meanwhile, the diversity along with the predominant sequence type, especially in Egypt, remains unknown. The purpose of the current study was to determine the diversity of the predominant MRSA clone ST239-MRSA (n = 50) isolated from different hosts and clinical samples and to illustrate the correlation between the resistance patterns, toxin genes, and the genetic background in Port-said and El-Sharkia Governorates, Egypt. The ST239-MRSA clone was analyzed by phenotypic antibiotyping and various genotypic assays comprising SCC*mec*, *agr*, *spa*, *coa*, and *coa*-RFLP in addition to toxin gene profiles. Most of the analyzed strains (40/50, 80%) were multidrug resistant (MDR), belonged to SCC*mec*-III, *agr*-I, and *coa* genotype I, and harbored *sea* and *pvl* genes. A negative correlation between the toxin gene profiles and antimicrobial resistance was recorded. Meanwhile, the correlation between the toxin gene profiles and the genetic background was not observed in this study. Although ST239-MRSA strains belonged to a single sequence type, they exhibited a high degree of phenotypic and genotypic diversity, indicating weak clonality and adaptability. With such diversity, it is assumed that these strains may have undergone different evolutionary processes during transmission events among and/or within a single host or tissue niche.

## Introduction

Methicillin-resistant *Staphylococcus aureus* (MRSA) has traditionally been considered a global pathogen (Gallagher et al., 2017). Indeed, most of the available studies have described the genetic diversity of MRSA strains belonging to multiple clones; meanwhile, few reports concerning a single clone have been reported. The proportions of both hospital and community-associated (CA) infections caused by single clones like ST239-MRSA strains have been increasing steadily in the past decades since the 1970s (Monecke et al., 2018). The ST239 is one of the dominant MRSA clones overtime worldwide, notably in Egypt (Abd El-Hamid et al., 2019), and it has been widely reported in Asia, Europe, the Middle East, e.g., Iran (Fatholahzadeh et al., 2009; Goudarzi et al., 2016; Sedaghat et al., 2018), and South and North America (Monecke et al., 2011). In terms of genetic structure, ST239-MRSA is an interesting clone as it harbors six of the seven multilocus sequence typing (MLST) housekeeping genes identical to ST8. However, ST239 and its clone relatives ST240 and ST241 differ from ST8 clone in *arcC* allele (*arcC*-2 in the former instead of *arcC*-3 in the latter) (Robinson and Enright, 2004).

Genetic typing is warranted to enable a better understanding of the infection dynamics. The typing methods used to determine the epidemiology of MRSA are sequence‐based methods such as MLST and staphylococcal protein A (*spa*) typing and non‐sequence‐based typing methods, which are based on staphylococcal cassette chromosome *mec* (SCC*mec*) elements, accessory gene regulator, *agr* (Magray et al., 2011) alleles, and restriction fragment length polymorphism (RFLP) patterns of coagulase (Coad et al., 2016) gene (*coa*-RFLP). Moreover, pulsed-field gel electrophoresis has been proposed as the gold standard for molecular epidemiological typing of MRSA bacterial strains (Ostojić, 2008). These methods have been recommended for determining the origin and the clonal relations and are used for enhanced discrimination of the closely related ST239-MRSA strains (Kong et al., 2017).

Similar to other MRSA clones, the ability of the ST239-MRSA clone to produce toxins and to resist certain antimicrobials affords a survival advantage and fitness under adverse conditions. Various sequence type (ST) clones of MRSA often display different antibiotic resistance and toxin patterns. Indeed, ST239-MRSA has been reported to be multidrug resistant (MDR), posing serious public health concerns (Kong et al., 2017). Another fitness feature of ST239-MRSA is the ability to produce several toxins, mainly staphylococcal enterotoxins (SE)A, K, and Q (*sea*, *sek*, and *seq*) and, to a lesser extent, toxic shock syndrome toxin (TSST), exfoliative toxins A and B (ETA and ETB), and Panton–Valentine leucocidin (PVL) leading to harmful toxic effects to the host (Xie et al., 2011; Boswihi et al., 2016; Alfouzan et al., 2019). However, many questions remain unanswered with regard to the correlation between the existence of toxin genes and antimicrobial resistance traits and many other factors such as the bacterial species, virulence, antimicrobial resistance, the ecological niche, and the host (Beceiro et al., 2013). Previous studies have indicated that some toxigenic strains belonged to a particular molecular type of MRSA (Baba et al., 2002; Chen et al., 2018). This implies that the molecular characteristics of MRSA strains affect their toxin gene profiles (Baba et al., 2002).

Although ST239-MRSA has been observed in several African countries (Kirchhelle, 2018); unfortunately, the molecular characteristics and virulence profiles of these strains are less clear in previous studies, and there are insufficient data on their epidemiological trends. Molecular epidemiological studies of clinical ST239-MRSA strains often depend on the application of different typing methods that produce various type assignments. Therefore, the present study aimed to investigate the molecular epidemiology and diversity of the ST239-MRSA clone belonging to different infected hosts and clinical sample types using a combination of typing approaches. Given the important consideration for planning and implementing the epidemiologic studies and outbreak investigations, we also aimed to evaluate the discriminatory abilities of the different typing methods and the strength of the correlation between them. The knowledge of the molecular epidemiology gained from this study would enable a better understanding of the characteristics of the ST239-MRSA clone and thus will be useful for the development of effective prevention and control measures.

## Materials and Methods

### Ethical Statement on Bacterial Strains

This is a case series study carried out to characterize some ST239-MRSA isolates during the period from June 2016 to August 2019. In this study, we investigated 105 *S. aureus* strains from both infected human (n = 60) and animal (n = 45) sources in Port-said and El-Sharkia Governorates, Egypt, including 30 previously identified strains as ST239-MRSA in one previous study for some of the co-authors (Abd El-Hamid et al., 2019). All human *S. aureus* strains associated with infections were kindly provided from different units in microbiology laboratories in Zagazig and Port-said University hospitals, which had acquired signed informed consents of the participating patients in this research. The human *S. aureus* strains were recovered from clinical samples including sputum (8), wound swabs (12), urine (14), pus (11), blood (9), cerebrospinal fluid (CSF) (4), and pericardial fluid (2). Meanwhile, *S. aureus* originating from milk samples of cows with mastitis were kindly supplied by the Department of Microbiology, Faculty of Veterinary Medicine, Zagazig University.

### Identification of *Staphylococcus aureus* Strains

The identification of all *S. aureus* strains was based phenotypically on standard bacteriological procedures including morphological, cultural, and biochemical characterization (Becker et al., 2015) in addition to using API 20S identification kit (bioMerieux, Marcy l’Etoile, France). Moreover, the investigated strains were confirmed using PCR analyses of *16S rRNA* and a species‐specific *nuc* genes (Abd El-Hamid and Bendary, 2015). The phenotypic identification of *S. aureus* strains carried out using standard bacteriological methods was based on mannitol fermentation on mannitol salt agar, β hemolysis on blood agar, the appearance of characteristic golden-yellow colonies, formation of Gram-positive grape-like clusters, and biochemical reaction results comprising the ability to coagulate rabbit plasma and the positive catalase reaction. For methicillin-resistance analysis, oxacillin and cefoxitin antibiotic susceptibility testing and PCR detection of *mecA* gene were performed (Abd El-Hamid and Bendary, 2015). The ST was characterized by MLST, which was based on seven housekeeping genes (*arc*C, *aro*E, *glp*F, *gmk*, *pta*, *tpi*A, and *yqi*L). Each sequence was then compared to the *S. aureus* MLST database (https://pubmlst.org/bigsdb?db=pubmlst_S.aureus_seqdef&page=profiles&scheme_id=1) to obtain an allele number as previously described (Enright et al., 2000).

### Antimicrobial Susceptibility Testing

Antimicrobial susceptibility of all ST239-MRSA strains was tested by disk diffusion method using the standard antimicrobial discs (Oxoid, Hampshire, UK) and according to the guidelines of Clinical and Laboratory Standards Institute (Clinical and Laboratory Standards Institute, 2019). Strain susceptibility was examined against 13 types of antimicrobials including oxacillin (OX; 1 μg), cefoxitin (FOX; 30 μg), ceftriaxone (CRO; 30 μg), imipenem (IPM; 10 μg), vancomycin (VA; 30 μg), clindamycin (DA; 2 μg), rifamycin SV (RF; 30 μg), trimethoprim‐sulfamethoxazole (SXT; 1.25/23.75 μg), erythromycin (E; 15 μg), ciprofloxacin (CIP; 5 μg), tetracycline (TE; 30 μg), gentamicin (CN; 10 μg), and chloramphenicol (C; 30 μg). The oxacillin minimum inhibitory concentration (MIC) values for all ST239-MRSA strains were determined by the broth microdilution method, and the results were interpreted according to the CLSI guidelines (Clinical and Laboratory Standards Institute, 2019). Moreover, according to the recommendations included in the CLSI document (Clinical and Laboratory Standards Institute, 2019), disk testing is not reliable for assessing vancomycin resistance. Therefore, to determine the susceptibility of ST239-MRSA strains to such antimicrobial agent confirming them as vancomycin resistant, the MIC of vancomycin (Sigma Aldrich, Darmstadt, Germany) was determined phenotypically using the broth microdilution method (Franklin et al., 2012) with twofold increasing concentrations of vancomycin from 0.0625 to 1024 µg/mL. Moreover, the vancomycin resistance among all investigated strains was confirmed by a multiplex PCR amplification of *van*A and *van*B genes using the previously reported primer sets and under the same thermal conditions reported previously (Abd El-Aziz et al., 2018).

The multiple antibiotic resistance (MAR) index for each strain was calculated as previously described (Krumperman, 1983) by dividing the number of antimicrobials to which the strain was resistant by the total number of antimicrobials to which the strain was exposed in the current study. Calculated values of MAR indices greater than 0.2 indicate high-risk sources of contamination, where several antibiotics are often used. Of note, the strains were defined as MDR if they were resistant to at least one agent in three or more antimicrobial categories used in this study (Magiorakos et al., 2012).

### Characterization of ST239-MRSA Genetic Background and Toxin Gene Profiles

All ST239-MRSA strains were analyzed for the detection of their genomic variations using uniplex PCR assays of *coa* and *spa* genes as stated previously (Hookey et al., 1998; Larsen et al., 2008). Digestion of *coa* PCR products with *Alu*I restriction endonuclease (Sigma, St. Louis, MO, USA) was then performed according to the manufacturer’s recommended protocol to monitor the variations in MRSA populations on the basis of the sequence variation within the 3′ end coding region of *coa* gene. Moreover, multiplex PCRs were performed for *agr* and SCC*mec* typing as described previously (Gilot et al., 2002; Zhang et al., 2005). Characterization of toxin gene profiles by detecting genes encoding nine enterotoxins (*sea*, *seb*, *sec*, *sed*, *see*, *seg*, *seh*, *sei*, and *sej*), exfoliative toxins (*eta* and *etb*), TSST (*tst*), and PVL (*lukSF-PV*) was performed using the previously reported PCR conditions (Mehrotra et al., 2000). All PCR analyses were carried out in triplicate. For the quality control of the toxin genes detection including PVL, *S. aureus* ATCC25923 and *Escherichia coli* ATCC25922 were included with all PCR runs as positive and negative controls, respectively. MRSA strains NCTC10442, N315, 85/2082, JCSC4744, and D12 were utilized as reference strains for detecting SCC*mec* types I, II, III, IV, and V, respectively. For each investigated gene, all PCR runs were performed with relevant PCR positive controls (DNAs from MRSA isolates previously confirmed to harbor sequences for any of the target genes by gel electrophoresis of PCR products). These positive controls were provided from the National Laboratory for Veterinary Quality Control on Poultry Production (NLQP), Animal Health Research Institute, Giza, Egypt. Additionally, sequencing of the amplicons of SCC*mec* and *lukSF-PV* genes was employed to confirm the genetic diversity of the ST239-MRSA clone according to the manufacturer’s protocol (Elim Biopharmaceuticals, Inc., Hayward, CA, USA). The resulting partial (433 bp) *lukSF-PV* gene sequences of ST239-MRSA strains were aligned with the fragments corresponding to a region measuring 2041.196–2041.58 kb in the genome of a selected *S. aureus* reference strain 14505 (GenBank accession number CP053640.1). The *lukSF-PV* gene segments had high nucleotide sequence identity with the reference sequence (100%). The sequencing reactions were carried out with single-stranded DNA templates. Prior to the sequencing of the SCC*mec* elements, the amplification products for SCC*mec* types were gel extracted and purified using the QIAquick Gel Extraction kit (Qiagen, Germany), and their nucleotide sequences were then determined. By utilizing the SCC*mec* Finder software found at https://bitbucket.org/genomicepidemiology/sccmecfinder.git, SCC*mec* element types were then identified. Of note, we followed the guidelines of the PCR unidirectional workflow.

### Bioinformatics and Statistical Analyses

Simpson’s index (Simpson, 1949) applied on the whole ST239-MRSA strains as well as on those per each host was used to reveal the diversity of the analyzed strains. Shapiro’s test (Royston, 1982) and Q-Q plot (Katayama et al., 2000) were used to assess and visualize the distribution of all variables, respectively. The correlation analyses were used to provide an estimate for the association among various variables, and then the correlation coefficient was visualized. The correlation analyses and visualization were done using R packages *corrplot*, *heatmaply*, *hmisc*, and *ggpubr* (Friendly, 2002; Galili et al., 2018; Jr, 2020). A heatmap supported by a hierarchical clustering dendrogram was generated to visualize the phenotype/genotype profiles of the examined strains. The analyses were conducted using the R environment (v. 3.6.2) (Kolde, 2018; Team, 2018). Fisher’s exact test was used to test whether the occurrence of resistance to certain antimicrobial differed significantly among studied hosts.

To assess the discriminatory power of the typing methods used in our study, the *D*-value of each typing method was assessed using Simpson’s index of diversity (Hunter and Gaston, 1988) calculating the probability that two unrelated strains sampled from the test population will be placed into different typing groups as follows:


$$D=1-\frac{1}{N\left(N-1\right)}\sum_{j=1}^{s}x_{j}\left(x_{j}-1\right)$$

where *D* is the index of discriminatory power, *N* is the number of unrelated strains tested, *S* is the number of different types, and *x_j_* is the number of strains belonging to the *j*th type assuming that strains will be classified into mutually exclusive categories. A *D*-value of 1.0 indicates that a typing method was able to distinguish each member of a strain population from all other members of that population; a *D*-value of 0.0 indicates that all members of a strain population were of an identical type. An index of 0.50 would mean that if one strain was chosen at random from a strain population, then there would be a 50% probability that the next strain chosen at random would be indistinguishable from the first.

## Results

### Multilocus Sequence Typing of *Staphylococcus aureus* Strains

According to the MLST results of 105 *S. aureus* strains, ST239 was proven for 50 strains. The detailed characteristics of all 105 strains are shown in **Table S1**. The 50 ST239 strains were recovered from 18 milk samples of cows with mastitis (40%) and 32 human patients (53.3%), including 6 sputum (75%), 6 wound swabs (50%), 6 urine (42.9%), 7 pus (63.6%), 4 blood (44.4%), 2 CSF (50%), and 1 pericardial fluid (50%). The different allelic sequences of the newly identified 20 ST239-*S. aureus* strains defined using the MLST website on the basis of the sequences of the internal fragments of seven housekeeping genes have been deposited in the GenBank with accession numbers MN880894:MN881033.

### Antimicrobial Resistance Profile of ST239-MRSA Clone

**Figure 1:**
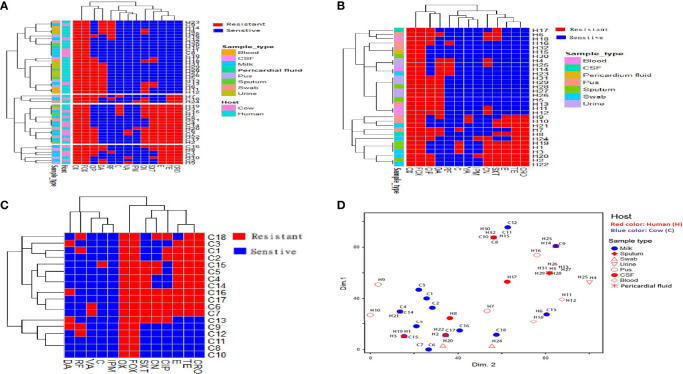
Distribution and hierarchical clustering of ST239 methicillin-resistant *Staphylococcus aureus* (MRSA) strains and the antimicrobial resistance variables based on the antimicrobial resistance profiles. **(A–C)** Heatmap showing the occurrence of antimicrobial resistance in the studied strains; all, human, and animal ST239 MRSA, respectively. Red and blue colors refer to resistance and sensitivity to a particular antimicrobial, respectively. The dendrogram represents the hierarchical clustering of the strains and antimicrobial resistance variables. The vertical lines dropped from the dendrogram branches show the 10 clusters that contained identical isolates. Different categories of hosts and sample types are color-coded on the right of the heatmap. **(D)** Non-metric multidimensional scaling analysis showing the overlap of ST239-MRSA strains belonging to different hosts and sample types based on antimicrobial resistance profile. CIP, ciprofloxacin; RF, rifamycin SV; TE, tetracycline; CRO, ceftriaxone; E, erythromycin; C, chloramphenicol; VA, vancomycin; DA, clindamycin; SXT, trimethoprim‐sulfamethoxazole; CN, gentamicin; IPM, imipenem; OX, oxacillin; FOX, cefoxitin.

Analysis of the antimicrobial resistance patterns of all ST239 *S. aureus* strains (n = 50) revealed that all of them (100%) were uniformly resistant to oxacillin and cefoxitin. All strains exhibited high-level resistance to oxacillin with MIC values >128 µg/mL. The existence of *mecA* gene in all examined ST239 strains proved that they were identified as MRSA. Irrespective of the host, the highest resistance of ST239-MRSA strains was against ciprofloxacin (54%). Meanwhile, relatively low resistance rates were observed against vancomycin (8%) and imipenem (4%) (**Table S2**). All strains derived from wound swabs were completely resistant to tetracycline, ceftriaxone, and gentamicin and sensitive only to vancomycin. Furthermore, all strains recovered from urine samples showed resistance to clindamycin and sensitivity to tetracycline, ceftriaxone, erythromycin, chloramphenicol, vancomycin, trimethoprim‐sulfamethoxazole, and imipenem (**Table 1**). With the use of Fisher’s exact test, it was found that only the occurrence of clindamycin resistance was considered different among the studied hosts. Four ST239-MRSA strains (8%) had vancomycin MIC of >16 μg/mL, and therefore, they were initially named vancomycin-resistant *S. aureus* (VRSA). Screening for *vanA* and *vanB* resistance genes revealed that 2 human (6.25%) and 2 animal (11.1%) strains harbored *vanA* or *vanB* genes confirming that they were all VRSA (**Table S3**). Interestingly, ST239-MRSA strains were resistant to at least 2–9 of the 13 antimicrobials generating MAR indices ranged from 0.15 to 0.69 (*D*-value = 0.86) with 24% of the strains showing a MAR index value of 0.30. However, only one isolate showed a MAR index of 0.23. High percentages of MDR were observed among our human (90.6%, 29/32) and cow (77.8% 14/18) strains. Antibiotyping of the examined strains revealed 26 different antimicrobial resistance patterns with *D*-value equal to 0.96 (**Figure 1A** and **Table S4**). The antibiotyping showed low discriminatory power for the human isolates (*D*-value = 0.937, **Figure 1B** **)** as compared to the animal ones (*D*-value = 0.967, **Figure 1C** **)**.

**Table 1:** Percentages of toxin genes and antimicrobials resistances of ST239-MRSA among different sample types.

Sample type (no.)	Toxin genes %							Antimicrobial resistances %										
	*pvl*	*sea*	*see*	*sec*	*tst*	*eta*	*etb*	CIP	RF	TE	CRO	E	C	VA	DA	SXT	CN	IPM
**Milk (18)**	39	44	6	0	6	33	11	39	22	61	61	61	6	11	22	50	39	5.6
**Sputum (6)**	67	33	17	0	17	0	0	50	0	50	50	50	17	0	33	50	33	0
**Wound swabs (6)**	100	50	0	0	0	0	0	67	17	100	100	67	33	0	17	67	100	17
**Urine (6)**	33	83	17	0	17	17	0	67	33	0	0	0	0	0	100	0	33	0
**Pus (7)**	57	29	29	14	0	14	0	57	14	43	43	29	14	29	57	29	14	0
**Blood (4)**	0	0	0	50	0	0	0	75	25	0	0	0	0	0	75	25	75	0
**CSF (2)**	0	0	0	50	50	0	50	100	0	50	50	50	0	0	50	100	0	0
**Pericardial fluid (1)**	0	0	100	0	100	0	100	0	0	0	0	0	0	0	0	0	0	0

CSF, cerebrospinal fluid; pvl, Panton–Valentine leucocidin; sea, see, and sec, staphylococcal enterotoxins A, B, and C; tst, toxic shock syndrome toxin; eta and etb, exfoliative toxins A and B; CIP, ciprofloxacin; RF, rifamycin SV; TE, tetracycline; CRO, ceftriaxone; E, erythromycin; C, chloramphenicol; VA, vancomycin; DA, clindamycin; SXT, trimethoprim‐sulfamethoxazole; CN, gentamicin; IPM, imipenem.

**Figure 2:**
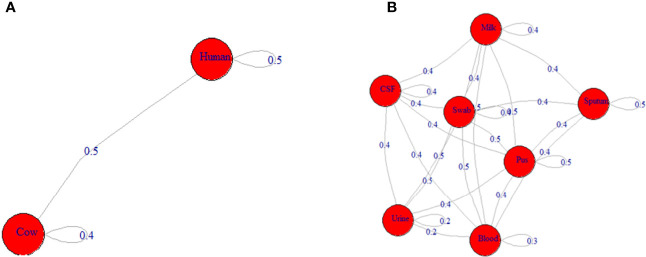
Network depicting the differences among ST239-MRSA strains based on the antimicrobial resistance profile. **(A)** The average distance among strains from humans and cows. **(B)** The average distance among strains from various samples.

Analysis of antimicrobial resistance patterns demonstrated a high variability among ST239-MRSA strains belonging to different hosts and from different sample types as evidenced by their large overlap revealed by the non-metric multidimensional scaling (nMDS) analyses (**Figure 1D**) with averaged binary distances equating to 0.4–0.5 among and within hosts (**Figure 2A**) and 0.2–0.5 among and within samples **(** **Figure 2B**), and averaged correlation coefficient, *r*(48) = 0.1–0.2 among hosts. It was evident that the antimicrobial resistance profiles of the strains colonizing the same host, particularly those within cows, were slightly more similar than those from the two hosts (i.e., considering cow and human strains together).

### Correlation Among Resistances to Different Antimicrobials

**Figure 3:**
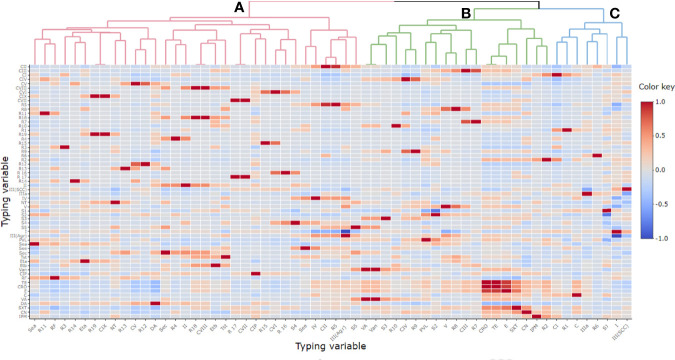
Pairwise correlation (*r*) among different variables of typing methods. Red and blue colors indicate positive and negative correlation, respectively. The color key refers to correlation coefficient (*r*). The darker colors imply stronger positive or negative correlations. Hierarchical clustering of the variables is shown as a dendrogram illustrating different clusters with different colors and letters (e.g., **(A–C)**. Variables that are identical among all strains are excluded and thus not shown in this figure. CIP, ciprofloxacin; RF, rifamycin SV; TE, tetracycline; CRO, ceftriaxone; E, erythromycin; C, chloramphenicol; VA, vancomycin; DA, clindamycin; SXT, trimethoprim‐sulfamethoxazole; CN, gentamicin; IPM, imipenem; CI-CIX, coagulase genotypes; R1–R19, *coa*-RFLP patterns; SCC*mec* (*II*, *III*, *IV*, and *V*), staphylococcal cassette chromosome *mec*; NT, non-typeable; *agr* (*I* and *III*), accessory gene regulator; S1–S5, *spa* PCR products; *van*, vancomycin resistance gene; *pvl*, Panton–Valentine leucocidin; *sea*, *see*, and *sec*; staphylococcal enterotoxins A, B, and C; *tst*, toxic shock syndrome toxin; *eta* and *etb*, exfoliative toxins A and B.

The correlation among individual antimicrobial resistance phenotypes was variable and showed an overall weak positive correlation, *r*(48) = 0.1. Ceftriaxone and tetracycline showed the highest positive correlation, *r*(48) = 1, while the clindamycin correlated negatively with tetracycline, *r*(48) = 0.4 (**Figure 3**).

### Molecular Typing of ST239-MRSA

**Figure 4:**
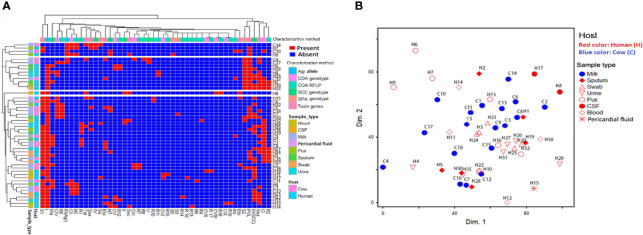
Distribution and hierarchical clustering of ST239-MRSA strains and the different genotypes based on different molecular typing methods. **(A)** Heatmap showing the occurrence of particular genotype/toxin gene in the studied ST239-MRSA strains. Red and blue colors refer to presence and absence of a particular feature, respectively. The dendrogram represents the hierarchical clustering of the strains and the genotype variables. Different categories of genotyping methods, sample types, and hosts are color-coded on the right of the heatmap. **(B)** Non-metric multidimensional scaling analysis indicating the presence of each subject in the 2-dimensional space and thus showing how the subjects could cluster or be separated from each other. CI–CIX, coagulase genotypes; R1–R19, *coa*-RFLP patterns; SCC*mec* (*II*, *III*, *IV*, and *V*), staphylococcal cassette chromosome *mec*; NT, non-typeable; *agr* (*I* and *III*), accessory gene regulator; S1–S5, *spa* PCR products; *van*, vancomycin resistance gene; *pvl*, Panton–Valentine leucocidin; *sea*, *see*, and *sec*, staphylococcal enterotoxins A, B, and C; *tst*, toxic shock syndrome toxin; *eta* and *etb*, exfoliative toxins A and B.

Coagulase genotyping revealed that ST239-MRSA strains presented diverse patterns with *D*-value equal to 0.79. Nine different coagulase genotypes (C^I^–C^IX^) were detected (**Figure 4A**). The CI (band size = 750 bp) was the predominant coagulase type being identified in 38% of the strains (19/50). Moreover, *coa*-RFLP was the best discriminatory method for investigating the ST239-MRSA strains, as it gave rise to 19 *coa*-RFLP patterns with a high *D*-value (0.92) (**Figure 4A**, **Table S5**).

Concerning the SCC*mec* typing, ST239-MRSA strains were discriminated into five types (*D*-value = 0.46) with SCC*mec*-III being the most common one (72%) (**Table S6**). The SCC*mec*-II, SCC*mec*-IV, and SCC*mec*-V types were detected among 3 human, 2 human, and 2 (one human and one animal) isolates, respectively. The carriage of these uncommon SCC*mec* types by these ST239 strains was also validated by DNA sequencing of their PCR products with accession numbers of MW563684:MW563719, MW563720:MW563722, MW563723:MW563724, and MW563725:MW563726 for SCC*mec*-III, SCC*mec*-II, SCC*mec*-IV, and SCC*mec*-V, respectively.

Grouping of *agr* alleles revealed only *agr* types I and III with *D*-value equal to 0.22 (**Table S6**). The *agr* type I was the most prevalent [88%, (44/50)]. All SCC*mec* type III strains (72%; 36/50) were of *agr* type I.

According to PCR amplification of *spa*-X region, the ST239-MRSA strains were grouped into 5 *spa* types with *D*-value equal to 0.53. The majority of the strains (64%, 32/50) were typed as S1 (400 bp). The S1 type was associated mainly with SCC*mec*-III and *agr*-I types being present in 71.9% of the strains (23/32) (**Figure 4A** and **Table S6**).

### Toxin Gene Profiles

Toxin gene profiles of ST239-MRSA strains revealed the presence of one or more toxin genes in 80% (40/50) of the tested strains. The most prevalent genes among the toxigenic strains were enterotoxins (75%) and *lukSF-PV* (57.5%) (**Figure 4A**). All strains derived from wound swabs possessed *lukSF-PV* gene (100%) (**Table 1**). The high prevalence of *lukSF-PV* gene among our isolates was confirmed by sequencing of the PCR products with accession numbers of MW570780:MW570802. Our study indicated that only one *et* allele (*eta* or *etb*) was detected in all ST239-MRSA strains. Of the nine enterotoxin genes examined, only *sea*, *sec*, and *see* were identified, and all the toxigenic strains possessed only one type of these genes. The most prevalent enterotoxin gene among the examined strains was *sea* (40%). The results also revealed that *lukSF-PV*, *see*, *sec*, and *tst* genes were more prevalent among the human strains as compared to the animal ones, but the reverse occurred with *sea*, *eta*, and *etb* genes. The combinative data of toxin gene profiles revealed 19 different toxin gene profiles with *D*-value equal to 0.91 (**Figure 4A**).

### Distribution of Different Methicillin-Resistant *Staphylococcus aureus* Genotypes Among Different Hosts and Sample Types

**Figure 5:**
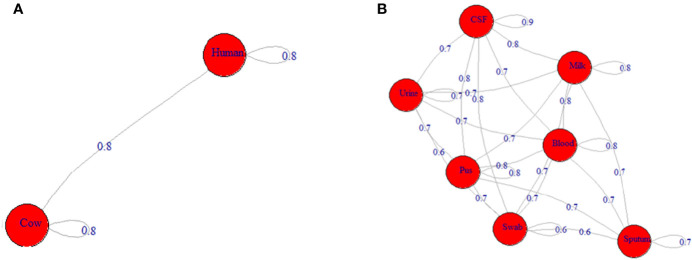
Network depicting the differences among ST239-MRSA strains based on both genotypes and toxin gene profile. **(A)** The average distance among strains from humans and cows. **(B)** The average distance among strains from various samples.

Depending on all molecular typing methods (*coa*, SCC*mec*, *agr*, and *spa*), the analyzed ST239-MRSA strains showed a considerable overlap (**Figure 4B**) with no segregation into defined clusters and with high averaged binary distance reaching 0.8 and low average positive correlation coefficient, *r*(48) = 0.2–0.3 among cow and human strains and between cow and human strains. The dissimilarity among strains of the same host was almost similar to that among different hosts (average binary distance = 0.78) (**Figure 5A**). Of note, the strains from the same sample were highly diverse with the range of averaged binary distance between 0.6 and 0.8. The CSF strains showed the highest heterogeneity (average binary distance = 0.8) and a very weak positive correlation (*r*(48) = 0.03), and the swab strains constituted the least diverse pool (average binary distance = 0.6) (**Figure 5B**). The homogeneity of the ST239-MRSA strains within each sample type and among different samples was quite similar, which was consistent for each of the molecular typing approaches. Moreover, the majority of ST239-MRSA strains have different genetic backgrounds when analyzed by combined molecular typing methods (**Figure 4A**). The *coa*-RFLP typing demonstrated the highest discriminatory power for strains within and among hosts in opposition to the *agr* typing.

### Correlation Among Antimicrobial Resistance Patterns, Molecular Typing Variables, and Toxin Gene Profiles

Considering all ST239-MRSA strains, the correlation analyses segregated all typing features into three main clusters (A, B, and C) distinguished by different colors as seen in the top of the dendrogram in **Figure 3** and each cluster contained a pool of typing parameters, each belonging to different typing methods. The majority of the typing methods (76.2%, 16/21) correlated to each other positively, and only five of them correlated negatively.

### Correlation Between the Existence of Antimicrobial Resistances and Toxin Genes

**Figure 6:**
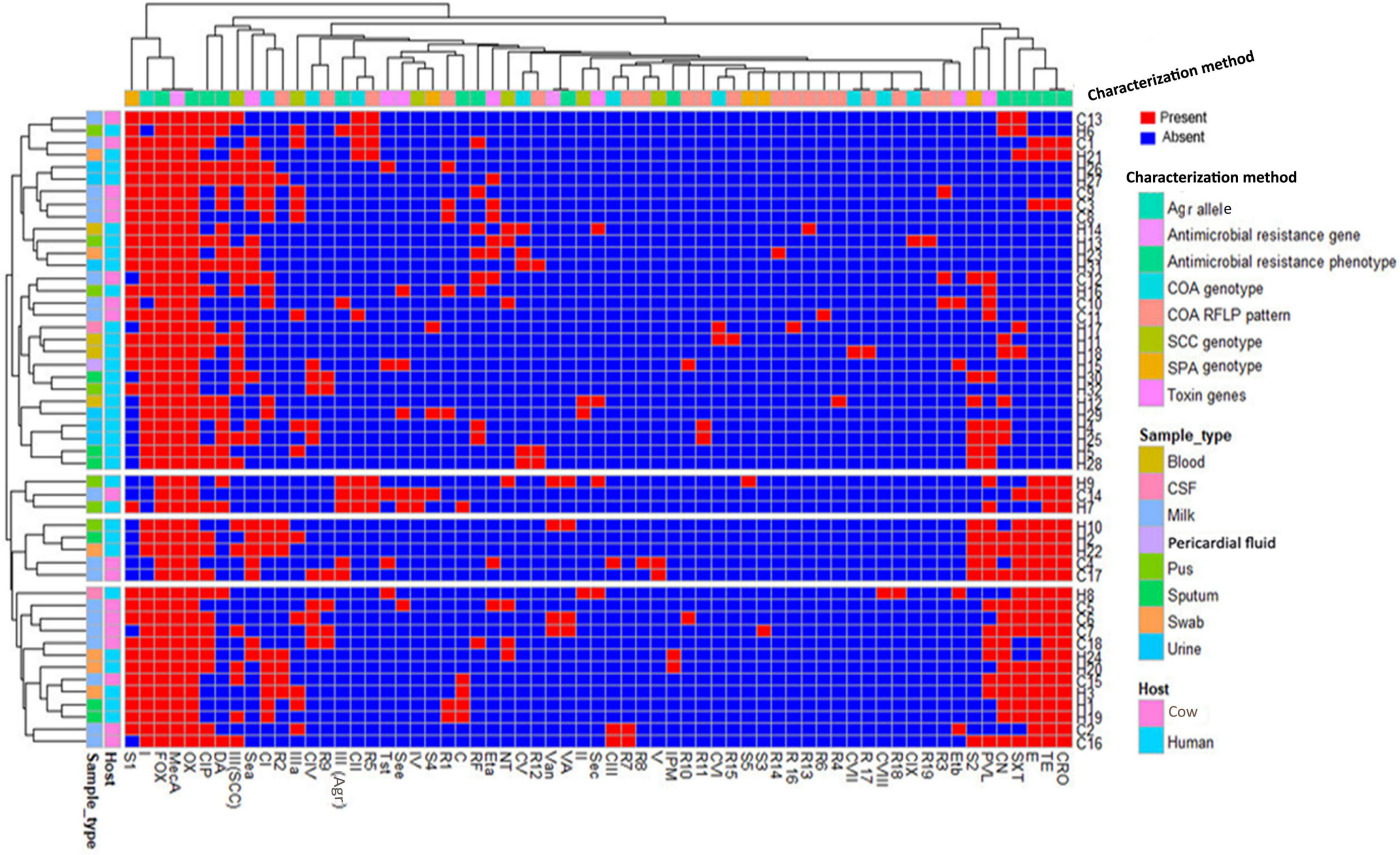
Heatmap showing the hierarchical clustering and overall distribution of ST239-MRSA strains based on their genotypes and antimicrobial resistance and toxin gene profiles. Red and blue colors refer to presence and absence of a particular feature, respectively. The dendrogram represents the hierarchical clustering of the strains and various features. Different categories of characterization methods, sample types, and hosts are color-coded on the right of the heatmap. CIP, ciprofloxacin; RF, rifamycin SV; TE, tetracycline; CRO, ceftriaxone; E, erythromycin; C, chloramphenicol; VA, vancomycin; DA, clindamycin; SXT, trimethoprim‐sulfamethoxazole; CN, gentamicin; IPM, imipenem; CI–CIX, coagulase genotypes; R1–R19, *coa*-RFLP patterns; SCC*mec* (*II*, *III*, *IV*, and *V*), staphylococcal cassette chromosome *mec*; NT, non-typeable; *agr* (*I* and *III*), accessory gene regulator; S1–S5, *spa* PCR products; *van*, vancomycin resistance gene; *pvl*, Panton–Valentine leucocidin; *sea*, *see*, and *sec*, staphylococcal enterotoxins A, B, and C; *tst*, toxic shock syndrome toxin; *eta* and *etb*, exfoliative toxins A and B.

Our study also revealed a negative correlation between the existence of toxin genes and antimicrobial resistances. Notably, MDR strains with maximum antimicrobial resistance patterns were associated with the least toxin gene profiles. Overall, 50% of toxigenic strains showed low MAR indices (≤0.38), but higher MAR indices (≥0.38) were observed in 80% of non-toxigenic strains (**Figure 6**).

### Association Between Genetic Background and Toxin Genes

The majority of PVL (78.3%, 18/23) and enterotoxins (83.3%, 25/30) positive strains belonged to *agr*-I genotype. Moreover, the majority of the strains harboring enterotoxin genes (56.7%, 17/30) were found to belong to SCC*mec*-III, and 38.9% (14/36) of ST239-MRSA-SCC*mec*-III strains possessed *sea*. Meanwhile, *sec* was found among ST239-MRSA SCC*mec*-II strains. The ST239-MRSA SCC*mec*-IV and SCC*mec*-V were found to carry *see* and *sea* genes, respectively. Furthermore, the majority of *lukSF-PV* positive strains (65.2%) belonged to SCC*mec*-III. All ST239-MRSA SCC*mec*-IV and SCC*mec*-V and the majority of non-typeable strains had more than one toxin gene. Moreover, the majority of toxin-producing ST239-MRSA strains belonged to *spa* type 1 (57.5%, 23/40) and *coa* type I (40%, 16/40) (**Figure 4A**).

## Discussion

The increase in the incidence of MDR bacterial and fungal infections has become an emerging threat to public health (Ghaith et al., 2020; Ghaly et al., 2020). The ST239-MRSA lineage is one of the most successful and persistent hybrid that showed high levels of drug resistance (Feil et al., 2008). Antimicrobial susceptibility testing of ST239-MRSA strains revealed the increasing prevalence of resistance to tetracycline, erythromycin, and ciprofloxacin as previously reported in China (Kong et al., 2017; Sedaghat et al., 2018). The haphazard use of antimicrobials to treat bacterial infections in animals often increases the resistance to these antimicrobials (Kirchhelle, 2018). The adapted ability of ST239-MRSA clone to enhanced antimicrobial resistance is extremely serious. Interestingly, more than 80% of our strains, especially of human origin, were MDR. This is consistent with a previous study conducted in India (Woodyard, 2011). Meanwhile, the MDR rate among human strains in our study (90.6%) is slightly lower than that previously reported in China (100%) (Kong et al., 2017). The indiscriminate use of antimicrobials in Egypt has rendered the commonly used antimicrobials completely ineffective in the treatment of this clone (Singh et al., 2016).

In terms of the susceptibility patterns of our MRSA isolates to the tested β-lactam antibiotics, all our investigated strains appeared resistant to oxacillin and cefoxitin; meanwhile, 48% and 4% of the isolates were resistant to ceftriaxone and imipenem, respectively. Consistent with a report from China, all ST239-MRSA strains were resistant to oxacillin and cefoxitin (Li et al., 2021), confirming the isolates as MRSA. Moreover, a previous research paper carried out in Israel revealed that 97.9% of MRSA isolates were sensitive to imipenem (Samba and Gadba, 1993). The highly mentioned susceptibility patterns to imipenem are reflective of its use as an effective antistaphylococcal agent against MRSA isolates as previously proven (Fan et al., 1986). However, a striking difference was detected in the susceptibility patterns of MRSA isolates to ceftriaxone. In Beijing, China, all isolates belonging to ST398-MRSA were resistant to ceftriaxone (Zhang et al., 2011). Additionally, Mushtaq et al. reported that only 16.7% of MRSA strains showed susceptibility to ceftriaxone (Mushtaq et al., 2017), confirming the misuse of ceftriaxone in these areas. Notably, the changing in the resistance profiles of MRSA clones to this antibiotic among various studies indicated that the traditional ceftriaxone-resistant MRSA isolates became less frequent with the emergence and spread of MRSA clones that were susceptible to this drug.

Currently, the emergence and spread of VRSA strains remain a challenging global health crisis. The clinical significance of VRSA is attributed to its potential to cause worldwide noticeable mortality in the absence of effective control and treatment options (Cong et al., 2020). Therefore, it crucially important to diagnose VRSA cases to facilitate its rapid isolation and recognition that help in implementing effective infection prevention and control practices. Interestingly, the current study revealed high resistance of human and animal isolates to vancomycin. In accordance with our study, some ST239-MRSA strains in Australia showed reduced vancomycin susceptibility (Holmes et al., 2014). Indeed, vancomycin resistance in ST-239 isolates has been rarely reported around the world and has not been reported previously in Egypt. Previous studies from Iran had indicated that a variant of ST-239 (named as ST1283 strain) was resistant to vancomycin (MIC of 512 μg/mL) (Azimian et al., 2012), and ST239-SCC*mec*-III/t037 from a university hospital showed resistance to vancomycin (Shekarabi et al., 2017). The latter clone was also reported to be resistant to vancomycin from hospitalized patients in Iran (Kouhsari et al., 2020). Similar to other bacteria, it is evidenced that the emergence of ST239 VRSA is a multifactorial phenomenon that could result from factors such as the intensive use of vancomycin in treating life-threatening infections (McDonald et al., 1997), bacterial genetic background, and yet-unknown evolutionary mechanisms. Therefore, there is a great challenge associated with the treatment of this clone, and this study further demonstrates the need to organize standard infection-control precautions to control the spread of vancomycin resistance in Egypt. Finally, these findings support the necessity for future surveillance studies on ST239-VRSA strains to keep their transmission to a minimum.

Concerning ciprofloxacin, clindamycin, and gentamicin, human strains showed higher resistance rates than animal ones, while the reverse occurred with tetracycline, ceftriaxone, and erythromycin. The continuous prescription of several Egyptian hospitals of ciprofloxacin, clindamycin, and gentamicin for chest infections (Eldeeb, 2006) and the large applications of tetracycline and erythromycin in veterinary fields (Zwjuang, 2005) lead to the variations in the resistance patterns to those antimicrobials among human and animal strains. The co-administration of erythromycin and tetracycline in the clinical field reflects the positive correlation between their resistances among our strains. Meanwhile, the differences in the mechanisms of resistance and genetic elements associated with the resistance to tetracycline and clindamycin (Fletcher and Macrina, 1991) may illustrate the negative correlation between these antimicrobials. The significant differences in the clindamycin resistance observed among our human and animal strains may be attributed to the absence of injection dosage form or the high cost, which limits the veterinary use of this antimicrobial in Egypt in comparison with other antimicrobials. Therefore, we confirm that the resistance pattern of the ST239-MRSA clone is well correlated with the extensive usage of antibiotics as announced in several reports (Havaei et al., 2017; Sedaghat et al., 2018)

Our results regarding the high prevalence of ST239-MRSA SCC*mec*-III agree with a previous report from India, which indicated that the majority (66.6%) of the ST-239 MRSA isolates belonged to SCC*mec*-III (Jain et al., 2019). Indeed, ST239-MRSA-SCC*mec* type III is considered to be an epidemic strain of hospital-associated MRSA in Asia (Chongtrakool et al., 2006). Moreover, ST-239 MRSA of the SCC*mec*-III is responsible for many outbreaks in healthcare settings all over the world and it is able to cause serious disseminated infections (Botelho et al., 2019). Moreover, a lower proportion of ST239-SCC*mec* type II (2%) was observed in a previous study conducted in Korea (Chong et al., 2013). Obviously, being the lineage with the highest prevalence in our isolates does not imply a general view on Egyptian ST-239 MRSA clone, as we did these analyses on only 50 isolates, and additional wide-scale analyses are needed to generalize these data.

Regarding the analysis of ST239-MRSA toxin gene profiles, our study indicated that nearly half of the strains were positive for *lukSF-PV* gene. In China, five identified ST239-MRSA isolates were all positive for *lukSF-PV* gene (Yu et al., 2008). Meanwhile, *lukSF-PV*-positive ST239-MRSA isolates were distinctly rare in many previous studies from various geographic areas (Alfouzan et al., 2013; Boswihi et al., 2016), suggesting that the carriage of *lukSF-PV* gene may be an unreliable marker for classifying the ST239-MRSA strains. Usually, the ST239-MRSA lineage lacks *tst* gene, while few studies have reported the presence of this gene in ST239 strains (Iwao et al., 2012; Kong et al., 2016) as was presented in our finding, where only few strains carried *tst* gene. Although previous reports (Xie et al., 2011; Jain et al., 2019) indicated the lack of *eta* gene in ST239 strains, it was determined to be the major exfoliative toxin type among our strains. The variable distribution of this virulent determinant was possible because it is located on a prophage that is integrated into *S. aureus* chromosome phages. To date, few ETA phages have been induced from bacterial cells and were found to be capable of transferring *eta* gene into *eta*-negative *S. aureus* strains, converting them into *eta* producers (Yoshizawa et al., 2000). Notably, *sea* was the most common enterotoxin gene detected among ST239-MRSA strains in previous studies (Tekeli et al., 2016; Jain et al., 2019). Similarly, our study showed that *sea* is the most prevalent enterotoxin gene among our ST239-MRSA strains.

Frequently, the MRSA strains with SCC*mec*-IV carry *lukSF-PV* gene encoding Panton–Valentine leucocidin (Bendary et al., 2016). Nevertheless, our results confirmed the association of *lukSF-PV* positive ST239 strains to SCC*mec*-III. This finding collaborated with an earlier report conducted in China (Yu et al., 2008), where five ST239 SCC*mec*-III isolates were PVL-positive. Taken together, the discrepancies between publications support that there is no absolute correlation between toxin profiles and the genetic background of this clone worldwide.

In the current analyses, it was expected that the ST239-MRSA strains belonging to different hosts (i.e., humans and cows) would exhibit more divergence compared to that among strains of the same host, and this was accepted phenotypically. Meanwhile, the diversity of both human and animal strains was almost similar to that among the same host depending on the genetic background. These results also suggest that the host is not the driving factor for the diversity of this clone, and possibly ST239-MRSA strains within the same origins may have undergone different evolution processes. The great diversity of these strains provides a wide array of defense strategies and challenges to both prevention methods and therapies (Yamamoto et al., 2012).

Despite the evolutions in the genetic background of the ST239-MRSA clone, the oldest truly pandemic MRSA strain, it is still the common and widespread clone. Of note, the mutation or the alteration in the housekeeping genes may lead to the emergence of new ST-MRSA clones as it was previously observed in *arcC*-allele leading to the emergence of ST239 from canonical ST8-MRSA clone. Unexpectedly, the ST239-MRSA clone can have genetic divergence without the emergence of a new ST-clone due to the conservation of the housekeeping gene sequences. It was previously reported that genetic divergence in this clone was related to the diverse geographic origins (Castillo-Ramírez et al., 2012). Therefore, the ST239-MRSA clone was clustered into 4 major clades including “European”, “Latin American”, “Turkish”, and “Asian” (Harris et al., 2010; Monecke et al., 2018). Surprisingly, in this study and in accordance with previous reports (Harris et al., 2010; Chen et al., 2014), the emerging ST239-MRSA subclones of one geographic origin and/or one host were announced. Therefore, the geographic origin and the host specificity were not the only driving factors for such genetic diversity. Possibly, the horizontal acquisition of the mobile genetic elements harbored antibiotic resistance genes, and virulence genes (Chen et al., 2014), clinical practice, prophage, and transgenerational adaptations were the major drivers alongside the geographic origin and host specificity for the genetic diversity within this clone, which may evolve and emerge as novel ST-MRSA clones in the near future. Crucially, ST239-MRSA clone has a selective advantage of the resistance provided by SCC*mec*-III; meanwhile, the prevalence of ST239 has recently declined, supporting the hypothesis that the recombination events, especially in the type of SCC*mec*, create a low fitness (Baltrus, 2013; San Millan and Maclean, 2017).

Our study revealed an overall negative correlation between the existence of antimicrobial resistance and the toxin genes. The toxigenic ST239-MRSA clone had a non-MDR profile, and they were susceptible to most antimicrobial agents, while MDR ST239-MRSA strains were less toxicogenic. This indicates the possibility of the acquisition of toxin genes on mobile elements at the expense of extended antibiotic resistance and vice versa. The consequence of these practices among ST239-MRSA strains may provide a fortuitous opportunity to correlate phenotypic antibiotic resistance patterns with the occurrence of toxin genes (Bendary et al., 2016).

The present study has some limitations. The lack of funds during the time of investigation precluded us from doing DNA sequencing for *spa* using the well-established *spa* typing method or whole-genome sequencing on the isolates, which are definitely the best validation approaches when it comes to validate their genetic diversity. However, we compensated for this via using PCR including positive and negative controls in addition to using sequencing techniques for unexpected results such as the high prevalence of *lukSF-PV* gene and the occurrence of different SCC*mec* genotypes. Additionally, the small number of strains used in this study hinders us to reach solid conclusions regarding the genetic characterization of the ST-239 isolates. Obviously, large isolate numbers should be used in future investigations to provide the information needed for infection control plans. Characterizing the current isolates and taking into consideration their origin [healthcare-associated (HA) versus CA] would be more interesting, yet the logistic difficulties of obtaining full data about the history of the patients, from whom the samples were taken, made this difficult and limited the scope of this study. In future investigations, applying whole-genome sequencing on the isolates would be appropriate to unravel strain diversity.

## Conclusions

The current study showed a high diversity of the ST239-MRSA clone possibly due to their recombination or evolutionary dynamism. Most strains were MDR and belonged to SCC*mec*-III, *agr*-I, *coa*-I (750 bp), and *spa*-I (400 bp) genotypes and harbored *sea* and *lukSF-PV* genes. These results have important implications, especially with regard to understanding the complex epidemiology of this clone, which is ultimately linked to strategies of its prevention and control. Therefore, there is an urgent need for more specific recommendations in terms of the control and prevention of such pathogens as well as their resistance such as applying more restricted isolation guidelines within each host/hospital unit and also avoiding the misuse of antibiotics, especially in veterinary fields. Future studies featuring a large sample size are needed to reach solid conclusions regarding more impacted infection control decisions.

## Data Availability Statement

The datasets presented in this study can be found in online repositories. The names of the repository/repositories and accession number(s) can be found in the article/**Supplementary Materials**.

## Ethics Statement

Ethical review and approval were not required for the study on human participants in accordance with the local legislation and institutional requirements. The patients/participants provided their written informed consents to participate in this study.

## Author Contributions

MB* and MA designed the study, carried out the antibiotyping and molecular analyses, and participated in the data analyses. MS performed the bioinformatics and statistical analyses and prepared the drafts and final version of the figures. MA-S, EK, MA, MB*, RM, and AS revised and discussed the figures, tables, and results. AS, HA, and WH wrote the primary version of the manuscript and participated in the design, antibiotyping, and molecular analyses. DG, HA, WA, and RM conceived the study and participated in the analysis. HR, MB and MG reviewed and edited the manuscript. All authors have read and agreed to the published version of the manuscript.

## Funding

MA-S extends his gratitude to the deputyship for research and innovation at the Ministry of Education in Saudi Arabia for supporting this work through project number 375213500.

## Conflict of Interest

The authors declare that the research was conducted in the absence of any commercial or financial relationships that could be construed as a potential conflict of interest.

## Publisher’s Note

All claims expressed in this article are solely those of the authors and do not necessarily represent those of their affiliated organizations, or those of the publisher, the editors and the reviewers. Any product that may be evaluated in this article, or claim that may be made by its manufacturer, is not guaranteed or endorsed by the publisher.

## Supplementary Material

The Supplementary Materials for this article can be found online at: https://www.frontiersin.org/articles/10.3389/fcimb.2022.782045/full#supplementary-material
