# Supplementary material for: Clonal Diversity and Epidemiological Characteristics of ST239-MRSA Strains
Source: Front Cell Infect Microbiol. 2022 Mar 25;12:782045. doi: 10.3389/fcimb.2022.782045 (PMC8990901; doi:10.3389/fcimb.2022.782045)
Supplement: Supplementary file 2 [file Table_2.docx]

| Isolates source (No) | No of resistant ST239-MRSA against antimicrobials (%) | | | | | | | | | | |
| --- | --- | --- | --- | --- | --- | --- | --- | --- | --- | --- | --- |
|  | CIP | RF | TE | CRO | E | C | VA | DA | SXT | CN | IPM |
| Milk (18) | 7  (38.9) | 4  (22.2) | 11  (61.1) | 11  (61.1) | 11  (61.1) | 1  (5.6) | 2  (11.1) | 4  (22.2) | 9  (50) | 7  (38.9) | 1  (5.6) |
| Sputum (6) | 3  (50) | 0  (0) | 3  (50) | 3  (50) | 3  (50) | 1  (16.7) | 0  (0) | 2  (33.3) | 3  (50) | 2  (33.3) | 0  (0) |
| Wound swabs (6) | 4  (66.7) | 1  (16.7) | 6  (100) | 6  (100) | 4  (66.7) | 2  (33.3) | 0  (0) | 1  (16.7) | 4  (66.7) | 6  (100) | 1  (16.7) |
| Urine (6) | 4  (66.7) | 2  (33.3) | 0  (0) | 0  (0) | 0  (0) | 0  (0) | 0  (0) | 6  (100) | 0  (0) | 2  (33.3) | 0  (0) |
| Pus (7) | 4  (57.1) | 1  (14.3) | 3  (42.9) | 3  (42.9) | 2  (28.6) | 1  (14.3) | 2  (28.6) | 4  (57.1) | 2  (28.6) | 1  (14.3) | 0  (0) |
| Blood (4) | 3  (75) | 1  (25) | 0  (0) | 0  (0) | 0  (0) | 0  (0) | 0  (0) | 3  (75) | 1  (25) | 3  (75) | 0  (0) |
| CSF (2) | 2 (100) | 0  (0) | 1  (50) | 1  (50) | 1  (50) | 0  (0) | 0  (0) | 1  (50) | 2 (100) | 0  (0) | 0  (0) |
| PC (1) | 0  (0) | 0  (0) | 0  (0) | 0  (0) | 0  (0) | 0  (0) | 0  (0) | 0  (0) | 0  (0) | 0  (0) | 0  (0) |
| Total (50) | 27 (54) | 9  (18) | 24 (48) | 24 (48) | 21 (42) | 5  (10) | 4  (8) | 21  (42) | 21 (42) | 21  (42) | 2  (4) |

**Table S2: Antimicrobial resistance patterns of ST239-MRSA strains isolated from different sources**

PC: pericardial fluid, CIP: ciprofloxacin, RF: rifamycin SV, TE: tetracycline, CRO: ceftriaxone, E: erythromycin, C: chloramphenicol, VA: vancomycin, DA: clindamycin, SXT: trimethoprim‐sulfamethoxazole, CN: gentamicin, IPM: imipenem, CSF: cerebrospinal fluid
