# Supplementary material for: Clonal Diversity and Epidemiological Characteristics of ST239-MRSA Strains
Source: Front Cell Infect Microbiol. 2022 Mar 25;12:782045. doi: 10.3389/fcimb.2022.782045 (PMC8990901; doi:10.3389/fcimb.2022.782045)
Supplement: Supplementary file 4 [file Table_4.docx]

**Table S4: Antimicrobial resistance profile and multiple antibiotic resistance indices of Egyptian 50 ST239-MRSA strains from different sources**

| **No** | | **Antimicrobial resistance profile** | **MAR index** | **Animal strains**  **(n=18)** | **Human strains, n=32**  **[Sample type (No)]** | **Total strains (n=50)** |
| --- | --- | --- | --- | --- | --- | --- |
| **1** | OX, FOX | 0.15 | 3 | 3 [Sputum (1), Pus (1), PC (1)] | 6 |  |
| **2** | OX, FOX, RF | 0.23 | 1 | 0 | 1 |  |
| **3** | OX, FOX, DA, RF | 0.31 | 2 | 1 (Blood) | 3 |  |
| **4** | OX, FOX, CIP, DA | 0.31 | 0 | 7 [Sputum (2), Urine (4), Pus (1)] | 7 |  |
| **5** | OX, FOX, CIP, RF | 0.31 | 0 | 1 (Pus) | 1 |  |
| **6** | OX, FOX, CIP, SXT | 0.31 | 0 | 1 (CSF) | 1 |  |
| **7** | OX, FOX, CN, DA, RF | 0.38 | 0 | 2 (Urine) | 2 |  |
| **8** | OX, FOX, CIP, CN, DA | 0.38 | 0 | 2 (Blood) | 2 |  |
| **9** | OX, FOX, CIP, CN, SXT | 0.38 | 0 | 1 (Blood) | 1 |  |
| **10** | CIP, TE, OX, FOX, CRO, E | 0.46 | 1 | 0 | 1 |  |
| **11** | DA, TE, OX, FOX, CRO, E | 0.46 | 1 | 0 | 1 |  |
| **12** | OX, FOX, CIP, CN, DA, SXT | 0.46 | 1 | 1 (Pus) | 2 |  |
| **13** | SXT, TE, OX, FOX, CRO, E | 0.46 | 2 | 1 (Sputum) | 3 |  |
| **14** | DA, TE, OX, FOX, CRO, E, VA | 0.54 | 0 | 1 (Pus) | 1 |  |
| **15** | SXT, TE, OX, FOX, CRO, E, VA | 0.54 | 0 | 1 (Pus) | 1 |  |
| **16** | CIP, RF, TE, OX, FOX, CRO, E | 0.54 | 1 | 0 | 1 |  |
| **17** | CIP, CN, TE, IPM, OX, FOX, CRO | 0.54 | 0 | 1 (Wound swab) | 1 |  |
| **18** | CIP, RF, CN, TE, OX, FOX, CRO | 0.54 | 0 | 1 (Wound swab) | 1 |  |
| **19** | CN, SXT, TE, OX, FOX, CRO, E | 0.54 | 1 | 0 | 1 |  |
| **20** | DA, CIP, TE, OX, C, FOX, CRO | 0.54 | 0 | 1 (Pus) | 1 |  |
| **21** | CN, SXT, TE, OX, C, FOX, CRO, E | 0.62 | 1 | 3 [Sputum (1), Wound swabs (2)] | 4 |  |
| **22** | DA, CIP, SXT, TE, OX, FOX, CRO, E | 0.62 | 0 | 1 (CSF) | 1 |  |
| **23** | CIP, CN, SXT, TE, OX, FOX, CRO, E | 0.62 | 1 | 2 [Sputum (1), Wound swab (1)] | 3 |  |
| **24** | CIP, CN, SXT, TE, OX, FOX, CRO, E, VA | 0.69 | 2 | 0 | 2 |  |
| **25** | CIP, CN, SXT, TE, IPM, OX, FOX, CRO, E | 0.69 | 1 | 0 | 1 |  |
| **26** | DA, CIP, CN, SXT, TE, OX, FOX, CRO, E | 0.69 | 0 | 1 (Wound swab) | 1 |  |

OX: oxacillin, FOX: cefoxitin, CIP: ciprofloxacin, RF: rifamycin SV, TE: tetracycline, CRO: ceftriaxone, E: erythromycin, C: chloramphenicol, VA: vancomycin, DA: clindamycin, SXT: trimethoprim‐sulfamethoxazole, CN: gentamicin, IPM: imipenem, MAR: multiple antibiotic resistance, PC: pericardial fluid, CSF: cerebrospinal fluid
