# Supplementary material for: Clonal Diversity and Epidemiological Characteristics of ST239-MRSA Strains
Source: Front Cell Infect Microbiol. 2022 Mar 25;12:782045. doi: 10.3389/fcimb.2022.782045 (PMC8990901; doi:10.3389/fcimb.2022.782045)
Supplement: Supplementary file 5 [file Table_5.docx]

**Table S5: Coagulase genotypes and *coa*-RFLP patterns of Egyptian ST239-MRSA strains from different sources**

| ***coa***  **genotype code** | ***coa*-PCR product (Approximate bp)** | ***coa*-RFLP**  **code** | ***coa*-RFLP pattern (Approximate bp)** | **Animal strains**  **(n=18)** | **Human strains, n=32**  **[Sample type (No)]** | **Total strains (n=50)** |
| --- | --- | --- | --- | --- | --- | --- |
|  |  |  |  |  |  |  |
| C^I^ | 750 | R1 | 750 | 3 | 4 [Sputum (1), Urine (2), Pus (1)] | 7 |
|  |  | R2 | 240, 410 | 2 | 6 [Sputum (1), Wound swabs (3), Urine (1), Pus (1)] | 8 |
|  |  | R3 | 400, 350 | 3 | 0 | 3 |
|  |  | R4 | 450, 250 | 0 | 1 (Blood) | 1 |
| C^II^ | 621 | R5 | 210 | 3 | 4 [Sputum (1), Pus (3)] | 7 |
|  |  | R6 | 350 | 1 | 0 | 1 |
| C^III^ | 556 | R7 | 190, 210 | 1 | 1 (Wound swab) | 2 |
|  |  | R8 | 210 | 1 | 0 | 1 |
| C^IV^ | 693 | R9 | 693 | 2 | 4 [Sputum (1), Wound swabs (2), Pus (1)] | 6 |
|  |  | R10 | 190, 210 | 1 | 1 (PC) | 2 |
|  |  | R11 | 210 | 0 | 2 (Urine) | 2 |
| C^V^ | 812 | R12 | 450, 250, 80 | 0 | 3 [Sputum (2), Urine (1)] | 3 |
|  |  | R13 | 450, 150 | 0 | 1 (Blood) | 1 |
|  |  | R14 | 550, 250, 80 | 1 | 0 | 1 |
| C^VI^ | 648 | R15 | 450, 250 | 0 | 1 (Blood) | 1 |
|  |  | R16 | 648 | 0 | 1 (CSF) | 1 |
| C^VII^ | 723 | R17 | 723 | 0 | 1 (Blood) | 1 |
| C^VIII^ | 410, 621 | R18 | 210 | 0 | 1 (CSF) | 1 |
| C^IX^ | 812, 913 | R19 | 450, 250 | 0 | 1 (Pus) | 1 |

*coa*: coagulase, bp: base pair, RFLP: restriction fragment length, PC: pericardial fluid, CSF: cerebrospinal fluid
