# Supplementary material for: Clonal Diversity and Epidemiological Characteristics of ST239-MRSA Strains
Source: Front Cell Infect Microbiol. 2022 Mar 25;12:782045. doi: 10.3389/fcimb.2022.782045 (PMC8990901; doi:10.3389/fcimb.2022.782045)
Supplement: Supplementary file 6 [file Table_6.docx]

**Table S6: Distribution of different genotypes of Egyptian ST239-MRSA strains from different sources**

| **SCC*mec***  **genotype** | ***agr* allele** | ***spa* type** | ***coa* genotype code** | **Animal strains**  **(n=18)** | **Human strains, n=32**  **[Sample type (No)]** | **Total strains (n=50)** |
| --- | --- | --- | --- | --- | --- | --- |
|  |  |  |  |  |  |  |
| II | I | S1 | C^V^ | 0 | Urine (1) | 3 |
|  |  |  | C^VIII^ | 0 | CSF (1) |  |
|  |  | S2 | C^I^ | 0 | Blood (1) |  |
| III | I | S1 | C^I^ | 5 | 6 [Pus (1), Urine (2), Wound swabs (2), Sputum (1)] | 36 |
|  |  |  | C^II^ | 4 | Pus (1) |  |
|  |  |  | C^III^ | 1 | 0 |  |
|  |  |  | C^IV^ | 1 | 2 [PC (1), Pus (1)] |  |
|  |  |  | C^V^ | 1 | 0 |  |
|  |  |  | C^VI^ | 0 | Blood (1) |  |
|  |  |  | C^VII^ | 0 | Blood (1) |  |
|  |  | S2 | C^I^ | 2 | 2 [Pus (1), Sputum (1)] |  |
|  |  |  | C^III^ | 0 | 1 (Wound swab) |  |
|  |  |  | C^IV^ | 0 | 3 [Sputum (1), Urine (2)] |  |
|  |  |  | C^V^ | 0 | Sputum (2) |  |
|  |  | S3 | C^IV^ | 1 | 0 |  |
|  |  | S4 | C^I^ | 0 | Urine (1) |  |
|  |  |  | C^VI^ | 0 | CSF (1) |  |
| IV | III | S1 | C^II^ | 0 | Pus (1) | 2 |
|  |  | S4 | C^II^ | 0 | Sputum (1) |  |
| V | III | S2 | C^III^ | 1 | 0 | 2 |
|  |  |  | C^IV^ | 0 | Wound swab (1) |  |
| NT | I | S1 | C^I^ | 0 | Wound swab (1) | 7 |
|  |  |  | C^IV^ | 1 | Wound swab (1) |  |
|  |  |  | C^V^ | 0 | Blood (1) |  |
|  |  |  | C^IX^ | 0 | Pus (1) |  |
|  | III | S1 | C^I^ | 1 | 0 |  |
|  |  | S5 | C^II^ | 0 | Pus (1) |  |

SCC*mec*: staphylococcal cassette chromosome *mec*, NT: non-typeable, *agr*: accessory gene regulator, *spa*: *S. aureus* protein A, S1-S5: *spa* PCR products of approximately 400, 450, 320*,* 200 and 500 base pairs, respectively, *coa*: coagulase, CSF: cerebrospinal fluid, PC: pericardial fluid
